# Long-Term Stability and Safety of Orthognathic-Orthodontic Correction for Skeletal Anterior Open Bite: A Systematic Review and Evidence Appraisal

**DOI:** 10.7759/cureus.98816

**Published:** 2025-12-09

**Authors:** Abdulrahman H Obeisi, Meshari M Alanazi, Suzan B Natto, Hatim A Alsalmi, Asma A Alzahrani, Raghad A Alotaibi, Abdullah A Aljari, Soltan B Alzahrani, Fahad B Alamri, Abdulaziz F Mohammed Alghamdi, Hattan S Katib

**Affiliations:** 1 Maxillofacial Surgery, Van Yuzuncu Yil University, Van, TUR; 2 General Dentistry, Alexandria University, Alexandria, EGY; 3 Periodontics, Al-Noor Specialist Hospital, Makkah Health Cluster, Ministry of Health, Mecca, SAU; 4 General Dentistry, King Abdulaziz University, Jeddah, SAU; 5 General Dentistry, Princess Nourah Bint Abdulrahman University, Riyadh, SAU; 6 General Dentistry, Sama Medical Center, Najran, SAU; 7 Dentistry, Al Baha University, Al Bahah, SAU; 8 Dental Medicine and Surgery, King Abdulaziz University Dental Hospital, Jeddah, SAU; 9 General Dentistry, King Abdulaziz Medical City, National Guard Hospital, Jeddah, SAU; 10 Dentistry, Specialized Dental Center, King Fahad Hospital, Madinah Health Cluster, Madinah, SAU

**Keywords:** cephalometric analysis, le fort i osteotomy, orthognathic surgery, skeletal anterior open bite, vertical stability

## Abstract

This systematic review evaluated the long-term stability and complication profile of combined orthodontic and orthognathic surgical treatment for managing skeletal anterior open-bite (AOB) in adolescents and adults. An electronic search of PubMed, the Cochrane Library, and Google Scholar identified English-language clinical studies (published from January 2004 to September 2025) that included at least one group treated with combined orthodontic and orthognathic surgical correction. Nonsurgical comparators such as skeletal-anchorage-based intrusion or orthodontic camouflage were also included when comparable outcomes were reported. Twenty studies, comprising predominantly retrospective cohort studies, met the eligibility criteria. Data extraction focused on study design, sample characteristics, surgical and orthodontic protocols, follow-up duration, cephalometric changes, relapse, complications, functional outcomes, and patient-reported measures. Risk of bias was assessed with Risk Of Bias In Non-randomized Studies, and certainty of evidence was summarized using Grading of Recommendations Assessment, Development and Evaluation.

Most surgical protocols used Le Fort I maxillary impaction alone or in combination with bilateral sagittal split osteotomy and counterclockwise mandibular rotation. Across the included studies, AOB was consistently converted to a positive overbite, with mean improvements and long-term preservation of the initial skeletal correction. Skeletal-anchorage-based molar intrusion achieved comparable overbite closure through posterior intrusion and mandibular autorotation, with a reduction in lower anterior facial height and limited incisor elongation. The reported relapse rates were frequently associated with persisting myofunctional habits and unfavorable baseline skeletal patterns. Complications were generally infrequent and mild; the most common findings were transient temporomandibular symptoms or neurosensory disturbances, and serious or life-threatening events and reoperations were rare. The overall certainty of evidence was mainly moderate for stability and cephalometric outcomes and low for complications due to methodological limitations. Within these constraints, combined orthodontic and orthognathic surgery appears to provide reliable functional and esthetic improvement for skeletal AOB, with skeletal anchorage representing a less invasive alternative in carefully selected adult patients.

## Introduction and background

Anterior open-bite (AOB) is a challenging malocclusion to manage because of its multifactorial etiology and high relapse rates. Characterized by the absence of vertical overlap between the maxillary and mandibular incisors when the posterior teeth are in occlusion, AOB may arise from skeletal, dentoalveolar, and functional discrepancies [1]. Epidemiological data suggest that AOB may affect up to 17% of young orthodontic patients, with population-based studies reporting prevalence values between 1.5% and 11% in children and adolescents, as well as higher rates in certain ethnic groups, particularly individuals of African descent, compared with European populations [2-4]. Clinically, skeletal AOB is often associated with Class II or Class III tendencies, increased mandibular and palatal plane angles, augmented lower anterior facial height, short posterior facial height, increased maxillary molar dentoalveolar height, lip incompetence, and mandibular retrusion or protrusion [5,6]. In this context, cephalometric variables such as the sella-nasion to mandibular plane angle (SN-MP) and the palatal plane to mandibular plane angle (PP-MP) are commonly used to describe vertical skeletal relationships, with higher values typically indicating a more hyperdivergent, open-bite-prone pattern. Similarly, changes in anterior overbite measured in millimeters translate into clinically visible differences in incisor contact and anterior guidance, such that even relatively small increases can be meaningful for both function and facial esthetics.

Multiple etiological factors have been implicated in the development and persistence of AOB, including hereditary skeletal patterns, altered soft-tissue morphology, neuromuscular dysfunction, and deleterious oral habits such as thumb sucking and tongue thrust [4-6]. In addition to functional impairment, patients frequently report difficulties with speech, mastication, and facial aesthetics, which may negatively affect oral health-related quality of life and psychosocial well-being [7]. Contemporary assessment increasingly incorporates patient-reported outcome measures (PROM) to capture changes in satisfaction, self-perceived appearance, and oral health-related quality of life following correction of skeletal AOB [8]. Findings from orthognathic surgery populations indicate that such patient-reported outcomes often parallel improvements in occlusion and facial esthetics, underscoring their relevance when evaluating treatment success [8]. These clinical and psychosocial burdens highlight the need for treatment strategies that not only correct the AOB but also deliver stable, long-term results.

A wide spectrum of management options has been used for AOB, ranging from orthopedic and orthodontic approaches in growing patients to combined orthodontic and orthognathic surgical protocols in skeletally mature individuals [1,5,6]. In children and adolescents, functional and fixed appliance therapies are used to redirect growth and dentoalveolar development, sometimes with adjunctive techniques such as posterior build-ups to promote anterior bite closure [5,6]. In contrast, treatment in nongrowing patients is substantially more demanding due to the limited ability to modify skeletal growth. In adults with established skeletal discrepancies, combined orthodontic and orthognathic surgery typically involving Le Fort I maxillary impaction with or without bilateral sagittal split osteotomy (BSSO) is widely regarded as the gold standard for achieving stable correction of skeletal AOB [7-9].

Despite advances in surgical planning and fixation, open-bite relapse remains a major concern. Systematic reviews have shown variable vertical stability following different orthognathic approaches for skeletal AOB, with outcomes influenced by surgical technique, fixation method, and posttreatment retention [9]. Relapse has been associated with several factors, including inadequate retention, persistent or recurrent oral habits, unfavorable growth and remodeling, and residual skeletal disharmony [9,10]. In addition to relapse, orthognathic surgery carries a recognized risk of complications, such as neurosensory disturbances, unfavorable splits, infection, bleeding, sinus complications, temporomandibular joint (TMJ) symptoms, and hardware-related issues, which may occur at different stages of the postoperative course [9,10].

Over the past two decades, advances in three-dimensional imaging and virtual surgical planning have refined the way skeletal AOB is analyzed and treated. Cone-beam computed tomography and three-dimensional photographic records allow more precise visualization of maxillofacial structures, simulation of maxillary impaction and mandibular rotation, and prediction of soft-tissue responses to surgery. At the same time, a more systematic collection of functional outcomes and patient-reported measures provides a broader framework for judging long-term success, extending beyond traditional cephalometric endpoints alone.

Several narrative and systematic reviews have addressed different aspects of AOB management, including prevalence, quality of life, vertical stability after surgery, and relapse following different treatment modalities [2,9,10]. However, there is still a need for a focused synthesis of the long-term skeletal and dental stability and the spectrum of complications specifically following combined orthodontic and orthognathic surgical management of skeletal AOB in adolescents and adults. This systematic review, therefore, aims to evaluate the long-term outcomes and complications associated with combined orthodontic-surgical treatment of skeletal AOB. In Population, Intervention, Comparator, Outcome (PICO) terms, the review considers adolescents and adults with skeletal AOB malocclusion (population), treated with combined orthodontic-orthognathic surgery (intervention), compared, where available, with alternative surgical or nonsurgical modalities such as skeletal anchorage or orthodontic camouflage (comparison), and assesses long-term skeletal and dental stability and complications as the primary outcomes. It also aims to summarize the certainty of the available evidence to inform clinical decision-making.

## Review

Materials and methods

PICO Framework

This systematic review was conducted following the Preferred Reporting Items for Systematic Reviews and Meta-Analyses guidelines [11]. An electronic search was done on PubMed, the Cochrane Library, and Google Scholar for articles published from January 2004 to September 2025. The PICO framework was followed:

Population (P): Adults and/or adolescents with a diagnosed skeletal AOB malocclusion.

Intervention (I): Combined orthodontic and orthognathic surgical correction as a two-phase treatment, with presurgical orthodontics to decompensate the dentition and align the arches, followed by orthognathic surgery (most commonly a Le Fort I maxillary impaction osteotomy, with or without a BSSO of the mandible) to correct the skeletal basis of the malocclusion.

Comparator (C): To be considered eligible, studies had to include at least one group treated with combined orthodontic and orthognathic surgery. Comparator groups could include surgery-only cohorts, surgery compared with temporary anchorage device (TAD)-supported orthodontic therapy as a nonsurgical alternative, or orthodontic camouflage protocols, as long as the reported stability and/or complication outcomes were the same.

Outcome (O): The primary outcome was long-term skeletal and dental stability, measured as anterior overbite (mm) after a minimum 12-month follow-up period (post-debond and/or postsurgery, depending on the study). Time points were prespecified as T1 (pretreatment), T2 (postoperative or post-debond), and T3 (long-term follow-up). Relevant cephalometric variables included overbite, SN-MP, Frankfort-Mandibular Plane Angle, PP-MP, and Upper First Molar to the Palatal Plane to assess skeletal and dental changes over time.

Inclusion Criteria

Eligible studies were clinical human articles published in English that included at least one group treated with combined orthodontic and orthognathic surgery for skeletal AOB malocclusion, involved adolescents or adults with a diagnosed skeletal AOB, and were published between January 2004 and September 2025. A minimum follow-up of 12 months after completion of active treatment and/or surgery was required, with overbite and/or cephalometric stability reported. Acceptable designs included prospective or retrospective cohort studies, comparative studies, or interventional series (with or without nonsurgical comparator groups such as TAD intrusion or camouflage).

Exclusion Criteria

Studies were excluded if they investigated AOB of purely dental origin (no skeletal component), did not include any orthognathic surgery group (e.g., purely orthodontic or functional appliance therapy without a surgical arm), were conducted exclusively on patients with craniofacial anomalies (e.g., cleft lip/palate, syndromic craniofacial conditions), or were nonclinical studies (in vitro or animal), narrative reviews, case reports, or expert opinions. These inclusion and exclusion criteria are summarized (Table 1).

**Table 1:** Inclusion and exclusion criteria for study selection TAD: temporary anchorage device

Category	Inclusion criteria	Exclusion criteria
Language	Articles published in English	The article was published in a language other than English
Population	Adolescents or adults with a diagnosed skeletal anterior open-bite malocclusion	Studies conducted exclusively on patients with craniofacial anomalies (e.g., cleft lip/palate, syndromic craniofacial conditions)
Condition/type of open bite	Skeletal anterior open-bite malocclusion treated as the primary condition of interest	Studies investigating anterior open bite of purely dental origin (no skeletal component)
Intervention	Clinical human studies including at least one group treated with combined orthodontic and orthognathic surgery for skeletal anterior open-bite malocclusion	Studies that did not include any orthognathic surgery group (e.g., purely orthodontic or functional appliance therapy without a surgical arm)
Publication period	Studies published between January 2004 and September 2025	Studies published prior to January 2004
Follow-up	Minimum follow-up of 12 months after completion of active treatment and/or surgery, with overbite and/or cephalometric stability reported	Less than 12 months after completion of active treatment
Study design	Prospective or retrospective cohort studies, comparative studies, or interventional series (with or without nonsurgical comparator groups such as TAD intrusion or camouflage)	Nonclinical studies (in vitro or animal), narrative reviews, case reports, or expert opinions

Search Strategy

A comprehensive search strategy was developed and implemented across the three databases searched in this review. The search incorporated a combination of Boolean operators (AND, OR) to capture relevant studies. The keywords used in the search strategy included the following: (“anterior open bite” OR “skeletal open bite”) AND (“orthognathic surgery” OR “Le Fort I” OR “segmental Le Fort I” OR “maxillary impaction” OR “posterior impaction” OR “bilateral sagittal split osteotomy” OR “BSSO” OR “sagittal split” OR “bimaxillary surgery” OR “surgery-first approach” OR “mandibular autorotation”) AND (“stability” OR “long-term” OR “relapse” OR “posttreatment” OR “retention” OR “complications”). The PICO framework and corresponding search terms used in the electronic database search are summarized (Table 2). The final electronic searches in all databases were completed in September 2025, and Google Scholar was used as a supplementary source by screening the most relevant hits until no additional eligible studies were identified.

**Table 2:** PICO framework and corresponding search terms used in the electronic database search BSSO: bilateral sagittal split osteotomy

PICO element	Search terms
Population (P)	“anterior open bite”, “skeletal open bite”
Intervention (I)	“orthognathic surgery”, “Le Fort I”, “segmental Le Fort I”, “maxillary impaction”, “posterior impaction”, “bilateral sagittal split osteotomy”, “BSSO”, “sagittal split”, “bimaxillary surgery”, “surgery-first approach”, “mandibular autorotation”
Comparator (C)	Comparator groups were defined at the eligibility level and could include surgery-only cohorts, temporary anchorage device (TAD)-supported orthodontic therapy as a non-surgical alternative, or orthodontic camouflage protocols, provided stability and/or complication outcomes were reported in a comparable way. No additional comparator-specific keywords were used in the electronic search
Outcome (O)	“stability”, “long-term”, “relapse”, “posttreatment”, “retention”, “complications”

Study Selection and Data Extraction

The identified articles were distributed between two reviewers for full screening. To ensure quality and relevance, the reviewers independently screened titles, abstracts, and full‐text articles to confirm that they met the inclusion criteria for combined orthodontic and orthognathic surgical correction of skeletal AOB malocclusion. Any disagreements were resolved through discussion or, if necessary, by a third reviewer.

For each included study, data were charted using a standardized extraction form that captured key details, including author, year, study design, sample size, follow-up period, and patient demographics (age and gender). Intervention data were extracted for the type of osteotomy performed, the magnitude and direction of skeletal movements, and whether nonsurgical orthodontic approaches or skeletal anchorage were used. Outcome data included cephalometric changes at different time points, relapse rates, hard- and soft-tissue complications, and any reported patient-reported or functional outcomes.

Study selection and data extraction were performed independently by two reviewers using a structured, prepiloted extraction form, with disagreements resolved by discussion or consultation with a third reviewer; no formal kappa statistic was calculated.

Data Synthesis

As the included studies were clinically and methodologically heterogeneous in terms of study design, surgical protocols, comparator treatments, outcome definitions, and follow-up duration, a quantitative meta-analysis was not undertaken. Instead, a descriptive narrative synthesis was performed. Continuous outcomes (e.g., overbite, cephalometric angles, and linear measurements) were summarized using the means and reported ranges provided in the original studies, and the results were grouped according to treatment modality, such as combined orthodontic-orthognathic surgery, skeletal-anchorage-based intrusion, or camouflage, where applicable. Where possible, findings were synthesized separately for stability, relapse, cephalometric changes, complications, esthetic outcomes, functional outcomes, and patient-reported measures, and interpreted in light of the overall risk of bias.

Relapse and stability outcomes were extracted according to each study’s original definition (for example, ≥1 mm loss of overbite or ≥2° increase in mandibular plane angle), and this heterogeneity in outcome definitions was a primary reason for using narrative synthesis rather than formal meta-analysis.

Risk of Bias Assessment

Risk of bias for nonrandomized studies was assessed using the Risk Of Bias In Non-randomized Studies (ROBINS-I) tool [12] across seven domains (confounding, selection of participants, classification of interventions, deviations from intended interventions, missing data, measurement of outcomes, and selection of the reported result). Two reviewers independently rated each domain and the overall judgment (low, moderate, serious), resolving disagreements by discussion with a third reviewer when required. Domain judgments were informed by prespecified confounders (baseline skeletal pattern, growth status, myofunctional habits, and retention protocol), clarity of intervention classification (surgical vs. skeletal anchorage vs. camouflage), and completeness/standardization of follow-up and outcome definitions. These ROBINS-I judgments fed directly into the Grading of Recommendations Assessment, Development and Evaluation (GRADE) certainty ratings for each outcome [13].

ROBINS-I domain judgments were based on prespecified criteria for confounding (baseline skeletal pattern, growth status, myofunctional habits, and retention protocol), follow-up completeness, and clarity of outcome definitions, and an overall "worst-domain" rule was applied when assigning the global risk-of-bias rating.

Results

Study Selection

**Figure 1:**
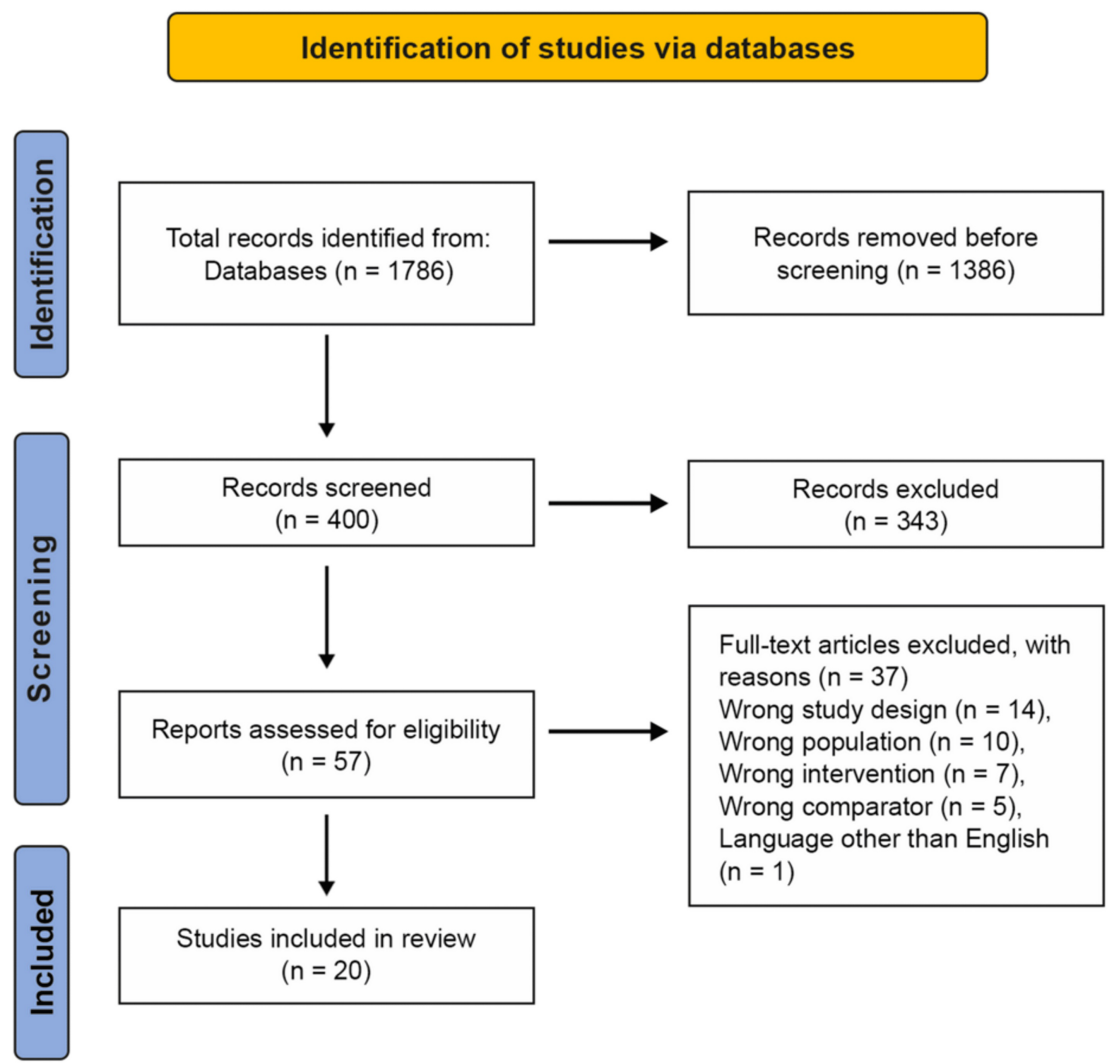
PRISMA flow diagram PRISMA: Preferred Reporting Items for Systematic Reviews and Meta-Analyses

In total, 1,786 records were identified through database searching. After removing 1,386 duplicates, 400 unique titles and abstracts were screened; 343 were excluded at this stage. Fifty-seven full-text articles were assessed for eligibility, of which 37 were excluded (wrong study design, n = 14; wrong population, n = 10; wrong intervention, n = 7; wrong comparator, n = 5; language other than English, n = 1). Twenty studies met the inclusion criteria and were included in the qualitative synthesis (Figure 1).

Study Characteristics

The 20 included studies [14-33] were predominantly retrospective cohort designs [14-29,31-33], with one prospective interventional study [30]. Sample sizes ranged from 10 to 57 patients and were drawn from populations in Europe, Asia, and the Americas. Most surgical cohorts underwent Le Fort I osteotomy alone or in combination with BSSO/bilateral sagittal split ramus osteotomy, often with counterclockwise rotation of the mandible and rigid internal fixation. Several comparative studies also included groups treated with skeletal-anchorage-based molar intrusion or orthodontic camouflage, allowing comparison between surgical and nonsurgical approaches in adults with skeletal AOB [17,23,30,31,33]. Key procedural details are summarized (Table 3).

**Table 3:** Characteristics of the included studies BSSO/BSSRO: bilateral sagittal split (ramus) osteotomy; BIVRO/IVRO: bilateral/intraoral vertical ramus osteotomy; RIF: rigid internal fixation; CCW: counterclockwise; PNS: posterior nasal spine; ANS: anterior nasal spine; MSLFI: multisegment Le Fort I; Me: menton; M-ILO: modified inverted L osteotomy

Study	Study design	Country	Sample size	Procedures	Maxillary movement	Mandibular movement	Fixation method
Maia et al. [14]	Retrospective	Brazil	39	LeFort I, BSSO, or both	Not specified	Not specified	Wire ligatures (64.1%), RIF (35.9%)
Teittinen et al. [15]	Retrospective cohort study	Finland and Switzerland	24	Maxillary impaction (Le Fort I) ± mandibular osteotomy (BSSO)	Not specified	Not specified	Rigid internal fixation
Arnett et al. [16]	Retrospective cohort study	Italy	30	MSLFI + BSSO	Not specified	Not specified	Plates with monocortical/unicortical screws, grafts
Chang et al. [17]	Retrospective comparative study	South Korea	42	G1: 2-jaw surgery (Le Fort I + SSRO/IVRO); G2: molar intrusion with miniscrews (nonsurgical)	Not specified	Not specified	Not specified
Kor et al. [18]	Retrospective cohort study	South Korea	29	Le Fort I + BSSRO ± genioplasty	3.39 mm (Group B)	~10 mm setback	4 L-shaped miniplates (maxilla), 4-hole miniplates + bicortical lag screws
Iannetti et al. [19]	Retrospective cohort study	Italy	40	Le Fort I ± BSSO	1.15-4.15 mm superior (incisor/molar)	3-3.43° posterior rotation (mandible)	2 miniplates (maxilla), 3 bicortical screws (mandible)
Torgersbråten et al. [20]	Retrospective cohort study	Norway	57	Le Fort I or BSSO (no bimaxillary cases)	Le Fort I: 4.1 mm impaction	BSSO: 8 mm advancement	Le Fort I: 2 L-shaped miniplates; BSSO: 3 bicortical screws ± miniplates
Espeland et al. [21]	Retrospective cohort study	Norway	40	1-piece Le Fort I osteotomy	PNS: 3.0 mm impaction; ANS: 1.2 mm inferior	Me: -1.8 mm (superior)	4 L-shaped miniplates (2 per side, 4 screws each)
Fontes et al. [22]	Retrospective cohort study	USA	31	BSSO with surgical closing rotation	Not applicable	3.7° closing rotation	Rigid internal fixation (bicortical screws ± miniplates)
Lee et al. [23]	Retrospective cohort study	South Korea	56 (19 intrusion; 37 surgery)	G1: maxillary molar intrusion with miniscrews (n = 19); G2: Le Fort I + BIVRO orthognathic surgery (n = 37)	3.2 mm (posterior impaction) (surgery group)	Mandible repositioned to maxilla (surgery group)	Rigid internal fixation (plates/screws)
Ooi et al. [24]	Retrospective cohort study	Japan	17	Group A: BSSRO; Group B: Le Fort I + BSSRO	Not specified	7.6° (A), 9.0° (B) counterclockwise rotation	Titanium miniplates
Ding et al. [25]	Retrospective longitudinal	Germany/China	10	Le Fort I + BSSRO (7/10 with 3-piece maxilla)	2.5 mm (posterior)	Mandible rotated 2.5° CCW	Maxilla: miniplates; mandible: wire osteosynthesis
Ellabban et al. [26]	Retrospective cohort study	UK	11	Le Fort I + BSSO with semirigid fixation	Mean: 4.1 mm (range 2-7 mm)	Mandible: ±6 mm (advancement or setback)	Synthes MatrixMidface and MatrixMandible plates
Aymach et al. [27]	Retrospective cohort study	Japan	12	M-ILO	None	CCW rotation only	Titanium locking miniplates
Stansbury et al. [28]	Retrospective cohort study	USA	28	BSSO with counterclockwise rotation	Not applicable	Mean: 6.1° rotation	Rigid internal fixation
Jensen and Ruf [29]	Retrospective cohort study	Germany	15	Le Fort I or bimaxillary surgery	Not specified	Not specified	Not specified
Akay et al. [30]	Prospective interventional	Turkey	10	Subapical corticotomy with skeletal anchorage (miniplates, zygoma screws; no Le Fort I/BSSO)	None	Mandibular CCW rotation	Titanium screws, miniplates
Nogueira et al. [31]	Retrospective comparative study	Brazil	39	G1: bimaxillary surgery with maxillary impaction; G2: orthodontic camouflage with/without extractions	Not specified	G1: mandibular advancement/setback; G2: none	Not specified
Wriedt et al. [32]	Retrospective cohort study	Germany	19	Bimaxillary osteotomy + fixed appliances	Not specified	Mandibular autorotation	Osteosynthesis plates
Kuroda et al. [33]	Comparative cohort study	Japan	23 (10 implant; 13 surgery)	G1 (implant): molar intrusion with skeletal anchorage; G2 (surgery): Le Fort I + IVRO/SSRO	3.0 mm (PNS)	Mandibular autorotation (implant); setback/advancement (surgery)	Miniplates, screws (implant); osteotomy (surgery)

Risk of Bias Assessment and Certainty of Evidence

Most included studies were retrospective cohort designs, which raises concerns about confounding, selection bias, and incomplete reporting. Outcomes such as overbite, cephalometric measurements, and complications were not always defined or recorded in a standardized way, and follow-up times varied across studies. These risks of bias were systematically evaluated using the ROBINS-I tool for nonrandomized studies, as shown in Table 4 [12]. In general, most studies were judged to have an overall moderate risk of bias, while five studies showed a serious risk of bias, mainly due to confounding or missing outcome data. Certainty of evidence was summarized using GRADE [13], overall ratings were moderate for overbite stability, relapse rate, cephalometric changes, comparisons of surgical protocols, esthetic outcomes, functional outcomes, molar/incisor relapse, facial height reduction, and patient satisfaction, while complication reporting had low certainty because of sparse and inconsistently collected data; no separate grading was undertaken for subgroup or sensitivity analyses.

**Table 4:** ROBINS-1 assessment of included studies D1-D7: ROBINS-I domains; D1: confounding; D2: selection of participants; D3: classification of interventions; D4: deviations from intended interventions; D5: missing data; D6: measurement of outcomes; D7: selection of reported result; ROBINS-I: Risk Of Bias In Non-randomized Studies

Study	D1: confounding	D2: selection	D3: intervention classification	D4: deviations	D5: missing data	D6: outcome measurement	D7: reported result	Overall risk of bias
Maia et al. [14]	Moderate	Moderate	Low	Moderate	Low	Low	Low	Moderate
Teittinen et al. [15]	Moderate	Moderate	Low	Moderate	Moderate	Low	Low	Moderate
Arnett et al. [16]	Moderate	Moderate	Low	Moderate	Moderate	Low	Low	Moderate
Chang et al. [17]	Moderate	Moderate	Low	Moderate	Low	Low	Low	Moderate
Kor et al. [18]	Moderate	Moderate	Low	Moderate	Moderate	Low	Low	Moderate
Iannetti et al. [19]	Serious	Moderate	Low	Moderate	Low	Low	Low	Serious
Torgersbråten et al. [20]	Moderate	Moderate	Low	Moderate	Low	Low	Low	Moderate
Espeland et al. [21]	Moderate	Moderate	Low	Moderate	Low	Low	Low	Moderate
Fontes et al. [22]	Moderate	Moderate	Low	Moderate	Low	Low	Low	Moderate
Lee et al. [23]	Moderate	Moderate	Low	Moderate	Low	Low	Low	Moderate
Ooi et al. [24]	Moderate	Moderate	Low	Moderate	Moderate	Low	Low	Moderate
Ding et al. [25]	Moderate	Moderate	Low	Moderate	Serious	Low	Low	Serious
Ellabban et al. [26]	Serious	Serious	Low	Moderate	Serious	Moderate	Moderate	Serious
Aymach et al. [27]	Serious	Serious	Low	Moderate	Serious	Moderate	Moderate	Serious
Stansbury et al. [28]	Moderate	Moderate	Low	Moderate	Moderate	Low	Low	Moderate
Jensen and Ruf [29]	Moderate	Moderate	Low	Moderate	Moderate	Moderate	Low	Moderate
Akay et al. [30]	Low	Low	Low	Low	Moderate	Low	Low	Low
Nogueira et al. [31]	Moderate	Moderate	Low	Moderate	Low	Moderate	Low	Moderate
Wriedt et al. [32]	Moderate	Moderate	Low	Moderate	Moderate	Moderate	Low	Moderate
Kuroda et al. [33]	Moderate	Low	Low	Low	Serious	Low	Low	Serious

A GRADE evidence profile was completed for all prespecified outcomes, with no separate grading undertaken for subgroup or sensitivity analyses (Table 5).

**Table 5:** Summary of GRADE certainty of evidence for key outcomes Certainty of evidence was graded using GRADE: ⬤⬤⬤⬤ = High; ⬤⬤⬤◯ = Moderate; ⬤⬤◯◯ = Low; ⬤◯◯◯ = Very low “Number of studies” reflects those reporting each specific outcome; effect estimates are narrative due to heterogeneity of measures “Surgery” denotes Le Fort I ± BSSO; “skeletal anchorage” denotes miniscrews/miniplates for molar intrusion. Downgrading reasons follow GRADE domains (risk of bias, inconsistency, imprecision, indirectness, and publication bias) SNB: sella-nasion-B point angle; MP-FH: mandibular plane-Frankfort horizontal; ANS-Me: anterior nasal spine-menton (lower anterior facial height); BSSO: bilateral sagittal split osteotomy; TMJ/TMD: temporomandibular joint/disorders

Outcome category	Number of studies	Effect estimate summary	Certainty of evidence	Reasons for downgrading
Overbite stability	14	Stability ranged from 64% to 100%; surgical and skeletal anchorage both showed improvement	⬤⬤⬤◯ Moderate	Retrospective designs, small samples, short follow-up, subgroup imbalance
Relapse rate	14	Relapse ranged from 6.7% to 37%; some bimaxillary and nonsurgical cohorts showed higher relapse, but results were heterogeneous	⬤⬤⬤◯ Moderate	Subgroup heterogeneity, inconsistent definitions, and limited power
Cephalometric changes	13	Improvements in SNB, MP-FH, ANS-Me, mandibular rotation, and incisor inclination tracked	⬤⬤⬤◯ Moderate	Small samples, retrospective tracking, no randomization
Surgical protocol comparisons	13	Le Fort I is generally more stable than BSSO; skeletal anchorage showed less molar relapse	⬤⬤⬤◯ Moderate	Non-randomized comparisons, small subgroups, and potential confounding
Complication reporting	6	No major complications; some TMJ symptoms and neurosensory issues post-surgery	⬤⬤◯◯ Low	Sparse data, retrospective recall, limited tracking
Aesthetic outcomes	2	The surgical group showed greater profile improvement than the camouflage group	⬤⬤⬤◯ Moderate	Subjective ratings, small effect size, no randomization
Functional outcomes	4	TMD improved postanchorage; trismus and sensory issues postsurgery	⬤⬤⬤◯ Moderate	Limited reporting, small samples
Molar/incisor relapse	3	Molar relapse is lower in the surgical group; incisor elongation is greater in the surgery group	⬤⬤⬤◯ Moderate	High variability, small sample, unclear clinical impact
Facial height reduction	4	A reduction of 3.6-4.0 mm was observed in both implant and surgical groups	⬤⬤⬤◯ Moderate	Consistent direction, but small samples and short-term data
Patient satisfaction	2	High satisfaction with occlusion and appearance (up to 95%)	⬤⬤⬤◯ Moderate	Self-reported outcomes, no control group

Publication Years

**Figure 2:**
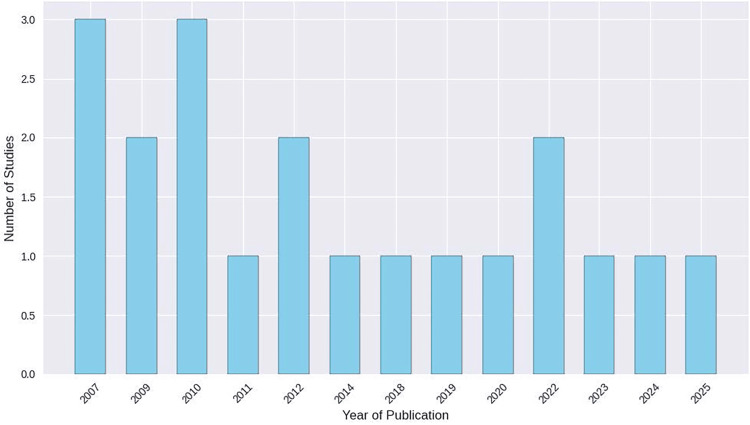
Histogram of publication years for the 20 included studies Image credit: This is an original image created by the author Abdulrahman H. Obeisi

To illustrate temporal trends in the evidence base, the distribution of publication years for the 20 included studies was plotted as a histogram (Figure 2).

The earliest cluster of publications occurred between 2007 and 2012, with 2007 and 2010 each contributing three studies, followed by additional single-center reports through 2014 and 2019. A second cluster of work appeared from 2019 to 2025, comprising studies published in 2022, 2023, 2024, and early 2025. This temporal distribution reflects both the historical development of orthognathic open-bite correction and the recent shift toward designs incorporating skeletal anchorage, longer follow-up periods, and functional outcome assessments.

Outcomes

In most cohorts, a positive overbite was found at the final follow-up, with mean improvements of approximately +1.3 to +7.0 mm, depending on the initial severity, choice of surgical protocol, and use of skeletal anchorage. Surgical approaches based on Le Fort I osteotomy, bimaxillary surgery, and mandibular counterclockwise rotation were dominant across the sample, and these were almost always used in combination with rigid internal fixation and comprehensive orthodontic treatment.

Cephalometric stability was often maintained, and several studies demonstrated limited changes in mandibular plane angle and vertical facial height during follow-up. Ding et al. [25] reported that around half to two-thirds of the original skeletal correction was preserved 15 years after surgery, while Espeland et al. [21] and Fontes et al. [22] described how dentoalveolar compensation (incisor extrusion and molar settling) helped maintain a positive overbite despite modest skeletal relapse. Studies using skeletal anchorage, such as Kuroda et al. [33] and Akay et al. [30], achieved open-bite closure primarily through posterior molar intrusion and mandibular autorotation, with reductions in lower anterior facial height and minimal reliance on incisor elongation.

The studies reported varying relapse rates, typically ranging from about 6.7% to 37% when relapse was defined as either ≥1 mm loss of overbite or ≥2° increase in mandibular plane angle. Functional habits such as mouth breathing, tongue thrust, and visceral swallowing were strongly associated with relapse in the long-term cohorts, such as in Wriedt et al. [32]. Baseline skeletal discrepancies, including isolated maxillary or mandibular inclination and increased vertical facial dimensions, were also linked with a higher risk of reopening in several reports.

Favorable esthetic outcomes were consistently reported, with surgical patients showing greater improvement in facial profile and smile esthetics than those treated with orthodontic camouflage. This pattern was especially clear in the comparative work of Nogueira et al. [31], where the bimaxillary surgery group demonstrated superior profile and smile scores compared with the camouflage group. Occlusal function improved in both surgical and nonsurgical groups, with bite force and occlusal contact area approaching or reaching control levels within approximately two years in the study by Lee et al. [23]. Similar functional gains were described in other cohorts using either skeletal anchorage or orthognathic surgery.

Complications

**Figure 3:**
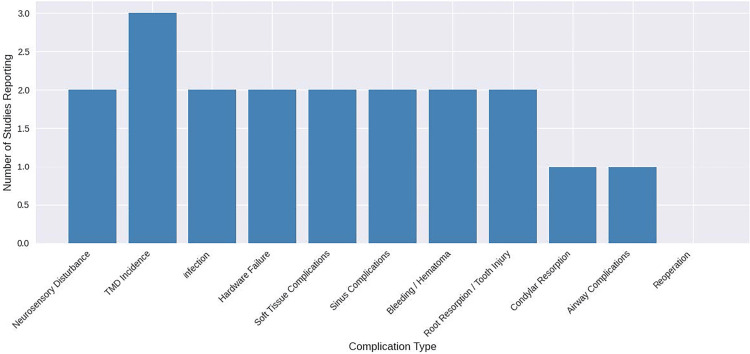
Frequency of reported complications across the 20 included studies TMD: temporomandibular disorder Image credit: This is an original image created by the author Abdulrahman H. Obeisi

To illustrate how often different adverse events were reported across the included cohorts, the frequency of each complication type was summarized in a bar chart (Figure 3).

Across the included studies, complications after combined orthodontic and surgical correction of skeletal AOB or skeletal-anchorage-based intrusion were generally infrequent and mild. A limited number of cohorts reported neurosensory disturbances; notably, Kuroda et al. [33] reported ≈10% of patients with altered sensation persisting greater than one year, and Wriedt et al. [32] reported hypesthesia in 5 out of 19 patients. TMJ symptoms were the most frequently reported complication, although these were often preexisting and tended to improve postoperatively, as observed by Maia et al. [14] and other surgical studies.

Other complications, including infection, hardware failure, soft-tissue or sinus-related problems, bleeding or hematoma, and root resorption or tooth injury, were reported in only one to three studies each and were typically transient, with no severe or permanently disabling cases documented. Condylar resorption was mentioned only once [16] and was asymptomatic. While single studies mentioned isolated reports of airway-related events and the need for reoperation, no study described life-threatening complications or the need for repeated major surgery.

Discussion

This systematic review evaluated the long-term outcomes and complications associated with combined orthodontic and orthognathic surgical correction of skeletal AOB malocclusion. Across 20 studies, the evidence consistently supports the effectiveness of this two-phase approach in achieving stable occlusal relationships, improved facial esthetics, and acceptable complication profiles in adolescent and adult populations [14-33]. All protocols achieved overbite correction, with mean improvements ranging from approximately +1.3 to +7.0 mm. Surgical modalities, particularly Le Fort I maxillary impaction, bimaxillary surgery, and mandibular counterclockwise rotation, were dominant and frequently paired with rigid internal fixation [14,16,18-22,24-29,31,32]. These approaches led to superior vertical control and skeletal stability, especially when combined with presurgical orthodontic decompensation [10]. Skeletal anchorage techniques, such as molar intrusion with miniscrews or miniplates, provided a less invasive alternative in selected cases and achieved comparable overbite correction with reductions in lower anterior facial height and limited incisor elongation [17,23,30,33]. However, most of the included studies assessed these outcomes using conventional two-dimensional cephalometry, whereas recent work has shown that three-dimensional imaging and surface-based analyses can reveal additional nuances in skeletal and soft-tissue change after orthognathic treatment [34-36].

Cephalometric stability was generally maintained, with several studies reporting only modest changes in mandibular plane angles and vertical facial dimensions over time. Notably, Ding et al. [25] demonstrated that approximately 50%-66% of the initial skeletal correction was preserved at 15 years, underscoring the potential for long-term success after bimaxillary surgery. Espeland et al. [21] and Fontes et al. [22] also reported that dentoalveolar compensation, particularly incisor extrusion and molar settling, helped to maintain a positive overbite despite some skeletal relapse. However, relapse rates varied widely (6.7%-37%) and were often linked to functional habits such as tongue thrust, mouth breathing, and visceral swallowing, as well as baseline skeletal disharmony [21,25,26,32]. Iannetti et al. [19] observed that patients with Class II skeletal patterns tended to show greater vertical relapse than those with Class III patterns, suggesting that the underlying anteroposterior relationship may influence long-term stability. Chang et al. [17] reported relapse ratios ranging from 31.6% to 82.8%, depending on the relapse definition used and the treatment subgroup; their findings highlight that even when overbite is restored initially, a substantial proportion of patients may experience partial reopening over time. Overall, these findings emphasize the importance of addressing myofunctional factors and ensuring adequate retention protocols posttreatment. They also highlight a methodological gap: few AOB cohorts have integrated three-dimensional volumetric or surface-based outcome measures, despite evidence that such techniques can provide a more comprehensive assessment of postsurgical change and stability [34,35].

Esthetic outcomes were consistently favorable in the surgical cohorts, with both single- and double-jaw procedures reporting improvements in facial profile and smile esthetics [16,21,26,27]. In the comparative study by Nogueira et al. [31], bimaxillary surgery produced greater improvements in profile and smile scores than orthodontic camouflage, supporting surgery as the preferred option for patients with pronounced skeletal discrepancies. Furthermore, functional recovery, including occlusal contact area and bite force, was achieved within approximately two years in cohorts treated with either skeletal anchorage or orthognathic surgery. This suggests that combined therapy can restore both form and function [20,23]. Complications were infrequent and generally mild. While temporomandibular dysfunction symptoms were the most frequently reported complications, they were often preexisting and tended to improve postoperatively in several series [14,20,21,23]. Neurosensory disturbances were observed particularly after mandibular procedures, but most resolved without additional intervention [16,24,28,32]. Only isolated cases of condylar remodeling or resorption were reported, and these were typically asymptomatic [16]. Few studies reported significant hardware failure, infection, or root resorption, and reoperations were rare or absent across the included cohorts. When interpreted alongside broader orthognathic surgery literature, which documents facial nerve and neurosensory risks as important but generally manageable elements of surgical morbidity [37], the present findings suggest that skeletal AOB correction does not appear to carry a uniquely elevated complication burden, although systematic, prospective complication recording is still lacking in this subgroup.

Despite these promising findings, the overall certainty of evidence was mostly moderate. Most studies were retrospective cohorts with small sample sizes and variable follow-up, and none employed randomized designs. Relapse was inconsistently defined, with definitions ranging from ≥1 mm overbite loss to ≥2° mandibular rotation, and outcome measures were heterogeneous, making direct comparison and pooling difficult [19,21,22,25-27,32]. Comparisons between different surgical protocols (e.g., Le Fort I vs. bimaxillary surgery, or surgery vs. skeletal anchorage) were based on nonrandomized designs with imbalanced subgroups, leaving residual confounding. Functional, esthetic, and patient-reported outcomes were insufficiently and inconsistently reported, and complication data were sparse and retrospective, limiting the precision of risk estimates. In contrast, systematic reviews of orthognathic surgery more broadly have shown that structured, validated PROM can capture large and sustained improvements in satisfaction, appearance, and oral health-related quality of life after surgery [8]. Accordingly, GRADE assessments rated the certainty as moderate for most stability and cephalometric outcomes and low for complication reporting. Future research should focus on prospective, multicenter studies with standardized outcome definitions, longer follow-up periods, and adequate power to compare surgical and nonsurgical protocols. Such studies should ideally incorporate three-dimensional imaging and virtual surgical planning to improve the accuracy and reproducibility of skeletal and soft-tissue measurements [34-36]. They should systematically use validated PROM instruments to align clinical, radiographic, and patient-perceived outcomes [8]. Generally, combined orthodontic and surgical correction remains the gold standard for managing skeletal AOB in nongrowing patients. When executed with appropriate planning, fixation, and retention strategies, it offers long-term skeletal and dental outcomes, high patient satisfaction, and a low complication burden. That said, addressing functional habits and optimizing retention protocols are critical to minimizing relapse and enhancing long-term stability.

Study Limitations

This review has several limitations that should be acknowledged. Most included studies were retrospective cohort designs, and this inherently increases the risk of selection bias and restricts causal inference. The generally small sample sizes reduced statistical power and introduced imprecision, particularly in subgroup comparisons. In addition, there was substantial variability in surgical protocols, fixation techniques, and orthodontic adjuncts, which limited comparability across studies. Relapse was inconsistently defined, ranging from ≥1 mm overbite loss to ≥2° mandibular rotation, making outcome synthesis challenging. Furthermore, follow-up durations were predominantly short- to medium-term, with relatively few studies extending beyond five years and only isolated cohorts with follow-up periods of 10-15 years [21,25]. None of the studies used randomized allocation, and blinding of outcome assessors was not reported.

Functional outcomes such as TMJ symptoms, bite force, occlusal contact area, and airway changes were infrequently reported and often lacked standardized measurements [20,23]. Similarly, esthetic and patient satisfaction outcomes relied on subjective evaluations without validated instruments or control groups, which may have led to overestimation of treatment benefits [26,27,31]. This stands in contrast to larger orthognathic surgery series in which validated satisfaction and quality-of-life instruments have been used systematically to document changes in patient-reported outcomes over time [8]. The reporting of complications was incomplete, with potential underreporting of minor or delayed issues, such as transient neurosensory disturbances, soft-tissue problems, and hardware-related events. In addition, most AOB cohorts did not use standardized complication taxonomies or structured neurological examinations, whereas dedicated orthognathic safety studies have shown that detailed, prospective assessment can identify specific risk patterns (e.g., for neurosensory or facial nerve disturbance) that may not be apparent in routine chart-based follow-up [37]. In some studies, different malocclusion classes and growth stages were combined within the same analysis; this may have introduced further heterogeneity and limited the applicability of pooled interpretations [19,20,32]. Another important limitation is that almost all included studies relied predominantly on two-dimensional cephalometric measurements. While these are widely used in clinical practice, they do not fully capture three-dimensional skeletal and soft-tissue changes, and may therefore underestimate or oversimplify postsurgical adaptations; emerging evidence suggests that three-dimensional imaging and surface-based analyses offer more comprehensive and reproducible evaluation of orthognathic outcomes [34-36]. Finally, this review was not prospectively registered in a database such as PROSPERO, which should be considered when interpreting the findings.

Due to these inconsistencies in methodology, study designs, interventions, and outcome measures, a quantitative meta-analysis could not be performed. The findings of this review should therefore be interpreted as a narrative synthesis rather than precise pooled estimates.

## Conclusions

Combined orthodontic and orthognathic surgical treatment remains a reliable option for managing skeletal AOB in nongrowing patients. Across the available evidence base, most patients achieve a positive overbite, restored function, and meaningful improvements in facial esthetics, with relapse occurring in a minority but clinically relevant proportion of cases. Skeletal-anchorage-based molar intrusion offers a less invasive alternative in carefully selected adults, providing comparable vertical control through posterior intrusion and mandibular autorotation.

Overall, long-term stability appears achievable when treatment planning is individualized and supported by appropriate fixation, retention, and management of myofunctional habits. However, the current evidence base is limited by predominantly retrospective designs, small sample sizes, heterogeneous protocols, and inconsistent outcome definitions. Future research should include well-designed prospective studies with standardized reporting, longer follow-up periods, and direct comparisons between surgical and nonsurgical protocols. These will help clarify prognostic factors and optimize treatment selection for skeletal AOB patients.
